# Visual Selection: Usually Fast and Automatic; Seldom Slow and Volitional

**DOI:** 10.5334/joc.13

**Published:** 2018-05-14

**Authors:** Jan Theeuwes

**Affiliations:** 1 Vrije Universiteit Amsterdam, Institute Brain and Behavior Amsterdam (iBBA), NL

**Keywords:** top-down and bottom-up control, attention, selection history

## Abstract

Recently it was argued that in addition to top-down and bottom-up processes, lingering biases of selection history play a major role in visual selection (Awh, Belopolsky & Theeuwes, 2012). Since its publication there has been a growing controversy about the terms top-down, bottom-up and selection-history in relation to visual selection. In the current paper we define these terms, discuss some controversies about these terms and explain what kind of effects should be considered to be the result of lingering biases of selection history, i.e., priming, reward/fear, and statistical learning. We discuss the properties of top-down selection (slow, effortful, and controlled) versus the properties of lingering biases of selection history (fast, effortless, and automatic). We adhere the position that the experience with selecting a particular feature or the location of a feature, may boost and sharpen its representation within the priority selection map above and beyond its physical salience. It is as if the experience may render a feature or location subjectively more salient. Our message of the current review is that true top-down control of visual selection occurs far less often than what is typically assumed. Most of the time, selection is based on experience and history. It is fast, automatic and occurs without much, if any, effort.

## Introduction

“Pay attention to the road” and “ignore those billboards” are typical examples of how we try to focus our attention on relevant information and ignore information that could distract us. We all are familiar with the act of paying attention to something relevant in the visual world. Paying attention is seen as a volitional act: just as we can decide to pick up a glass, we can decide to direct our attention to a particular object in space. In some cases, however, we may find ourselves looking at a bright flashing light even though we had no intention to do so. In that case, particular properties of stimuli (salience) impinging on the retina determine selection which may not be in line with our plans, goals and intentions.

The visual world we encounter in everyday life is complex and filled with a large amount of information. In order to act and behave in a goal-directed manner, we focus our limited resources on relevant information and filter out distracting information. Selective attention is the mechanism that determines what we see and act upon. Attentional selection has been considered to be the result of the interaction between intentions and the goals of the observer (current selection goals) and the physical properties of the visual environment (salience of the objects). All prominent models of attentional control have described attentional selection as the result of the interaction between bottom-up and top-down processes (Corbetta & Shulman, 2002; Itti & Koch, 2001; Theeuwes, 2010) sometimes referred as stimulus-driven and goal-driven selection (Egeth & Yantis, 1997; Ludwig & Gilchrist, 2002), exogenous and endogenous attention (Carrasco, 2011; Posner, 1978; 1980; Theeuwes, 1994a), or automatic and non-automatic control (Jonides, 1981; Shiffrin & Schneider, 1977). In a recent review, Awh, Belopolsky and Theeuwes (2012) pointed out that this classic theoretical dichotomy may no longer hold as in many cases attentional selection can neither be explained by current selection goals nor by the physical salience of potential targets. Awh et al. (2012) suggested a third category which they named “selection history” to stress that the history of attentional deployments can elicit lingering and enduring selection biases, unrelated to top-down goals or the physical salience of items.

**Figure 1:**
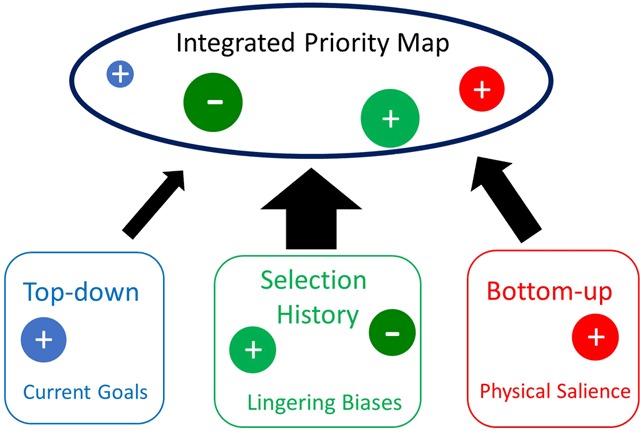
A schematic representation of a priority map that integrates three sources of selection bias: the observer’s current selection goals, selection history, and the physical salience of the items competing for attention (adapted from Awh et al., 2012).

*Current goals, physical salience* and *selection history* (see Figure 1) all feed into an integrated priority map which represents a conceptual framework accounting for selection priority, i.e., which object is selected next. The competition on this topographical map of space between the input from current goals, physical salience and selection history determines, in a winner-take-all fashion, the object that ultimately will be selected. Compared to the original Figure of Awh et al. (2012), there is also a minus added to the integrated priority map as recent studies have shown that selection history (in particular statistical learning regarding the location of the distractor) can also result in inhibition within the priority map (Wang & Theeuwes, 2018, submitted-a, b; Ferrante, in press). Locations that are inhibited compete less for attention than all other locations.

Even though this viewpoint of Awh et al. (2012) is shared by many others (see also Anderson, 2016; Chelazzi, Perlato, Santandrea, & Della Libera, 2013; Jiang, 2017; Todd & Manaligod, 2017, for similar ideas), the introduction of this representation has led to discussion about what is meant by top-down, by bottom-up and by history-based selection. The current paper provides a theoretical background about these terms and discusses special cases in which there seem to have been some controversies about these terms.

## Bottom-up attention (Physical Salience)

It is generally agreed that bottom-up attention is driven by the properties of the environment. The type of control is assumed to lie outside the organism: as soon as a specific stimulus occurs, attention is immediately directed to the stimulus. Posner (1980), who labeled this “exogenous attention” explained the attentional response towards the occurrence of a particular stimulus in terms of a physiological reflex. Neumann (1984) referred to bottom-up attention as automatic processes and stated that *“automatic processes are under the control of stimulation rather than under control of the intentions (strategies, expectancies, plans) of the person”* (Neumann 1984, p. 258). It is agreed that bottom-up attention biases the observer towards selecting stimuli that are salient; stimuli that stand out from the environment (Itti & Koch, 2001; Theeuwes, 2010). Several computational models have stressed the role of salience in attentional selection (Itti & Koch, 2001; Itti, Koch, & Niebur, 1998). These models basically take an image as input and process the image in parallel across various feature channels using different spatial scales. The end result is a set of topographic feature maps which are then combined into a saliency map (Koch & Ullman, 1985).

A paradigm that has been instrumental in the discussion about bottom-up attention is the additional singleton paradigm developed by Theeuwes in the early nineties (Theeuwes, 1990, 1991, 1992, 1994b). In this task, observers search for one specific and clearly defined salient target singleton while another distractor singleton, which is more salient but irrelevant to the task, is also present. Reaction time data showed that relative to a no-distractor baseline condition, the time to find the target increased when an irrelevant singleton was present. This result was explained in terms of bottom-up attention: before observers were able to respond to the target, the irrelevant singleton summoned attention against the intentions of the observers. If observers direct attention to objects even though they are trying to do otherwise, one speaks of attentional capture (Theeuwes, 1992, 2010a). Note that when the irrelevant color distractor was made less salient, its presence had no effect on search anymore (see Theeuwes, 1992, Exp. 3). On the basis of these findings, Theeuwes argued that initial selection was fully driven by physical saliency of the stimuli. The basic findings of the additional singleton paradigm has been replicated many times using reaction time (Bacon & Egeth, 1994; Geyer et al., 2008; Kim & Cave, 1999; Kumada, 1999; Theeuwes, 2004), *d*-prime (Theeuwes, Kramer & Kingstone, 2004; Theeuwes & Chen, 2005), saccadic eye movements (Godijn & Theeuwes, 2002; Ludwig & Gilchrist, 2002; Mulckhuyse, Van der Stigchel, & Theeuwes, 2009; Theeuwes et al., 1998; Theeuwes et al., 1999); Event-related Potentials (ERP; Hickey, McDonald & Theeuwes, 2006; Schubo, 2009) and hand movements (Hunt, von Muhlenen, & Kingstone, 2007).

When considering bottom-up attention two characteristics are important. First, *bottom-up attention is fast*. For example, several studies have used eye movements as the dependent measure. In these so called oculomotor capture tasks, the fastest eye movements go to the most salient element in the display (typically the distractor) while the slower eye movements go to less salient elements (typically the target) (Theeuwes et al., 1998, 1999; van Zoest, Donk & Theeuwes, 2004). Hickey, van Zoest and Theeuwes (2010) used the N2pc (an event-related potential index of (shifting) covert attention) as a dependent measure and showed that immediately following display onset, attention was directed to the salient irrelevant singleton, while slower shifts of attention were directed to the less salient target stimulus. In a study using directional manual responses using a joystick, it was shown that participants only moved the joystick to the distractor location when participants were forced to respond quickly (Hunt et al., 2007). Using a variant of the additional singleton task in monkeys showed that for the first 175 ms post-stimulus, the firing rate of neurons did not show any influence of the task set. In other words, whether a singleton was task relevant or irrelevant had no effect on the initial firing rate, suggesting that these early processes (<175 ms) are completely bottom-up (Ogawa and Komatsu, 2004).

Second, *bottom-up attention is involuntary*. In order to ensure that bottom-up attention is truly involuntary, it is important that the goal of the observer is orthogonal to the stimulus that captures attention. In many previous studies, the location of the stimulus capturing attention was uncorrelated with the location of the target. For example, Yantis & Jonides (1990) showed when the target presented among multiple no-onset items happened to be presented with abrupt onset, observers responded faster suggesting that the stimulus presented with abrupt onset captured attention. Yet, in that type of design, in which the stimulus capturing attention is uncorrelated with the target, attending to the onset does not hurt performance (because at chance level the onsetting item is the target). A better design is when the salient event and the target location never coincide, as is done in the additional singleton paradigm. In such a paradigm, attending to a distractor is never beneficial and hurts performance, because it is never the target. Only when one uses a design in which the top-down goal is orthogonal to the capturing features, one can establish whether bottom-up attention is involuntary.

## Top-down attention (Current Goals)

The definition of top-down attention is somewhat more ambiguous than that of bottom-up attention. Just as I can decide to pick up a glass of beer, I can decide to covertly direct my attention to the glass. Directing attention is a volitional act under control of the observer. It is important to stress the aspect of volitional control as it suggests that, at any given time, the observer is in charge of what to select. Almost all definitions about top-down control stress the aspect of volition. For example: “*a slower, top-down mechanism with variable selection criteria, which directs the ‘spotlight of attention’ under cognitive, volitional control*” (Itti & Koch, 2000; p. 1490); “*volitional shifts of attention are thought to depend on “top-down” signals derived from knowledge about the current task (e.g., finding your lost keys)*” (Buschman & Miller, 2007; p. 1860); “*Attention can also be voluntarily directed to objects of current importance to the observer*” (Connor, Egeth & Yantis, 2004; p. 850), “*voluntary orienting can be considered aspects of top-down attentional control*” (Hopfinger, Buonocore & Mangun, 2000, p. 284), “*Top-down visual attention is a voluntary process in which a particular location, feature, or object relevant to current behavioral goals is selected internally and focused upon*” (Katsuki & Constantinidis, 2014, p. 515) and “*volitional top-down process, which can exert its influence through acts of will*” (Baluch & Itti, 2011, p. 210).

In this respect, we assume that top-down control is similar to “taking an overt decision” comparable to when we initiate an overt voluntary action (see Pashler, Johnston & Ruthruff, 2001). It is nothing else than my decision to “pick up a glass of beer” or to “put one leg before the other”. In this respect, it can also be my decision to direct my attention to an object that is relevant to me (e.g., a glass of beer). From a neural point of view, top-down attention results in an increased neural activity for a particular location in space that is relevant for executing a particular task relative to all other locations that are not relevant.

The notion that observers are able to direct attention “at will” to a particular location in space dates back to Posner’s classic cueing tasks (Posner, Snyder & Davidson, 1980). In these studies, before display onset, observers receive a central symbolic cue (e.g., an arrow or a verbal instruction) indicating the likely location of the upcoming target (for example with 80% validity). This implies that on most trials, the central cue indicates the location where the target is likely to appear. The typical finding in these type of experiments is that observers are faster and more accurate when the target appears at the cued location than when it occurred at the non-cued location (see also Theeuwes, 1989). The interpretation of these results is that on each trial, observers use the cue to voluntarily direct spatial attention to the location indicated by the cue. It is important to realize that in these types of experiments, the location indicated by the cue varies randomly from trial to trial, which implies that on each trial, observers shift attention to the indicated location *at will*. Therefore, the endogenous Posner cueing task is one of the most prominent examples of top-down attention that is truly volitional. Being volitional, under voluntary control is seen as one of the most important aspects of top-down attention.

Some have claimed however that orienting in response to centrally presented arrow (as was originally done by Posner, 1980) is not necessarily top-down as an arrow pointing to a location in space has properties that evokes automatic orienting to the location indicated by the arrow (e.g., Ristic & Kingstone, 2006). Even though this may be the case when using arrows, there have been other ways to direct attention to a location in space which cannot be automatic. For example, Theeuwes and Van der Burg (2007, Experiment 1) presented as a central cue, a number which represented the hand of a conventional clock (e.g., the number 9 represented a location to the left of fixation, the number 12 the top location). With these arbitrary cues, Theeuwes and Van der Burg showed large validity effects of the cue on d-prime suggesting that, on each trial, participants intentionally directed their attention to one of the 12 locations in the display. Crucially, this location varied from trial to trial and observers were perfectly able to do it suggesting true volitional top-down attentional control.

A second characteristic of top-down control is that it is relatively slow. It takes time to implement top-down processes that allow a volitional guidance of attention. Shifts of pure endogenous attention in response to a centrally presented verbal cue as in the Posner cueing tasks take about 200 to 300 ms (Müller & Rabbitt, 1989) whereas exogenous shifts to peripheral onsets only take about 100 ms (Nakayama & Mackeben, 1989). It is generally assumed that top-down attention is slow because information from higher cortical areas needs to be sent to earlier areas in order to guide attentional control.

## Selection History (Experience-Based Selection)

As noted in addition to top-down and bottom-up attention, Awh et al. (2012) suggested a new category which was labelled “selection history”. The underlying notion is that attention is often deployed in a way that is neither consistent with the top-down goals of the observer, nor is it driven by salient stimuli impinging on the retina. In addition to top-down and bottom-up control, these lingering biases due to selection history affect the integrated priority map (see Figure 1). The underlying notion of what is meant by selection history is that through (explicit or implicit) learning (i.e., processes shaped by past episodes of attentional selection), particular stimuli may receive “value” that affects future selection episode above and beyond top-down and bottom-up factors. Currently, there are three broad classes of phenomena, that are related to lingering biases due to selection history (for a complete review, see Failing and Theeuwes, 2017).

### 1. Priming

When a stimulus (feature) is repeatedly attended in the previous trial, it is more efficiently selected and identified on the current trial. Maljkovich and Nakayama (1994), for example, demonstrated the influence of priming in the context of a search task (see also Hillstrom, 2000; Olivers & Humphreys, 2003). Priming between trials, or intertrial priming, occurred for up to eight successive trials, even when participants were unware of repetitions (Maljkovich & Nakayama, 2000), or when they were informed that the target was unlikely to be same between trials (Maljkovich & Nakayama, 1994). Overall, priming is considered to be low-level facilitatory effect on perceptual processing and is determined by traces of past selection history (Kristjansson, 2010; Kristjánsson & Campana, 2010; Lamy & Kristjansson, 2013; Theeuwes & Van der Burg, 2007, 2011; Theeuwes, 2013; Theeuwes, Reimann, & Mortier, 2006).

Hickey Chelazzi and Theeuwes (2010) also demonstrated the automatic nature of priming. In their study, when participants received a high reward, and on the next trial they had to search a target with the same color, they were faster than when the colors switched. Crucial for the present discussion however, is that in a follow-up experiment, participants were told that whenever a high reward was received, the colors associated with the target and the distractor would switch. In other words, if the target were red, and participants received a high reward they knew that in 80% of the trials, the color of the target on the next trial would be green. Even though it was detrimental to search and reward payout, participants could not switch their color set: they remained biased towards the color that gave high reward even though they knew that it would have been beneficial to switch to the other color. In a study by Theeuwes and Van der Burg (2013) it was shown why primed stimuli may draw attention. In their study, observers performed two tasks (temporal order judgment task (TOJ) and simultaneity judgement task (SJ)) in which observers had to determine the order in which two test stimuli were presented. For both tasks, the stimulus that was primed was seen earlier in time than a non-primed equally salient stimulus. It was concluded that through priming, one of the stimuli became more salient which accelerated its processing and thus caused prior entry into awareness.

Priming is an important driver of attentional selection and should not be mistaken for what has been called response priming, a selection bias that is the result of the buildup of automatic associations between stimuli and response tendencies. In all priming studies discussed (e.g., Maljkovic and Nakayama, 1994; Pinto et al., 2005; Theeuwes et al., 2006; Theeuwes & Van der Burg, 2007) the response of the participant is unrelated to what they are searching for. In this sense priming represents the efficiency with which the target can be selected; not the speed with which the response can be emitted (see Theeuwes and Van der Burg, 2007, 2011).

### 2. Reward/Acquired fear

Several studies have demonstrated that a stimulus can acquire value after it has been associated with reward during a training session (Anderson et al., 2011a, b; Bucker & Theeuwes, 2014; Della Libera & Chelazzi, 2006, 2009; Failing & Theeuwes, 2014, 2015, 2017; for a recent review see Failing & Theeuwes, in press). Crucially, this bias in attention occurs even when the stimulus is non-salient, task irrelevant and no longer predicts reward (Anderson, 2011b). The basic finding in all these studies is that a distractor stimulus that is associated with reward captures attention even when observers are instructed to search for the target. In other words, capture occurs independent of the top-down set of the observer. Crucially, this value-driven attentional capture is determined by the rate of learning as participants who demonstrated faster learning showed larger capture by the previously high rewarded stimulus during the test session (Jahfari & Theeuwes, 2017).

Other studies have demonstrated that a neutral stimulus can acquire value after it has been associated with fear (Schmidt et al., 2015, 2017; Preciado et al., 2017; Mulckhuyse & Dalmaijer, 2016). By means of fear conditioning, a neutral stimulus becomes associated with the delivery of an aversive event, such as an electric shock (CS+) while another stimulus is associated with no shock (CS–). As a consequence of this procedure, the fear-conditioned stimulus (CS+) captures attention more strongly than equally salient non-conditioned stimuli (CS–) (Mulckhuyse, Crombez & Van der Stigchel, 2013; Mulckhuyse & Dalmaijer, 2016; Schmidt et al., 2015, 2017) and biases attention such that the efficacy of sensory processing (measured in d-prime) is enhanced (Preciado, Munneke & Theeuwes, 2016). Also, threat signaling stimuli do not only capture attention, they can also capture the eyes (Nissens, Failing & Theeuwes, 2016) above and beyond physical salience (see also Hopkins et al., 2016 in which observers were not aware of the contingencies).

### 3. Statistical learning

It is well known that contextual information containing invariant properties of the visual environment can bias visual attention. Sensitivity to these statistical regularities makes it possible to interact more effectively with the visual world (Chun & Jiang, 1998). Several previous studies have focused on how statistical regularities regarding the target can bias selection. For example, the efficiency of finding the target can be improved when the target consistently appears at specific locations in previously seen displays relative to random locations (Chun & Jiang, 1999). Geng and Behrmann (2005) showed that targets presented in high probability locations are detected faster than those in low probability locations (see also Jiang, Swallow, Rosenbaum, & Herzig, 2013). Even though important, these findings may not be surprising because the target is task relevant and it is well-known that observers are capable of directing spatial attention to locations in space that are likely to contain a target (e.g., Posner, 1980).

Crucially however, recent studies have shown that statistical learning regarding items that are not relevant for the task (i.e., the location of the distractors) have a strong effect on attentional selection. For example, using the additional singleton paradigm, Wang and Theeuwes (2018) demonstrated that statistical regularities regarding the distractor location had a strong effect on attention (see also Ferrante et al., in press for a similar result). In Wang and Theeuwes, participants searched for a salient shape singleton while ignoring a color distractor singleton. Crucially, the color distractor singleton was presented systematically more often in one location than in all other locations. For this high-probability location, Wang and Theeuwes found that both attentional capture was reduced when the distractor appeared at the high probability location and selection of the target was less efficient when it happened to appear at this location. Crucially, most participants were not aware of the statistical regularities even though search was biased away from these high probability distractor locations (see also Zhao, Al-Aidroos, & Turk-Brown, 2013; Wang & Theeuwes, submitted-b).

Our notion is that statistical learning can induce plasticity with the spatial priority map such that locations that are likely to contain a target are boosted (“plus” sign in Figure 1) and locations that are likely to contain a distractor are inhibited (“minus” sign in Figure 1). The claim of inhibition or distractor suppression resembles a recent account referred to as the “signal suppression” hypothesis stating that through active inhibition it is possible to avoid capture by salient signals (Gaspelin and Luck, 2018). Crucially however while Gaspelin and Luck (2018) argue that this inhibition is top-down in nature, we claim that inhibition is solely driven by statistical learning.

## Controversies

Since the publication of the Awh et al., paper, there have been some discussion about whether particular effects should be labeled as bottom-up, top-down or experienced-based (see Klein, 2017). Here we discuss some of the issues that have led to these discussions.

### 1. If it is not bottom-up, it must be top-down

Because many researchers adhere the classic dichotomy between bottom-up and top-down control, some have argued that if it is not bottom-up, by definition it has to be top-down. As there is general consensus that bottom-up attention is driven by the physical properties of external stimuli, all processes that are not due to external stimulation must by definition be top-down. For example, Wolfe, Butcher, Lee & Hyle (2003) argued that priming is an example of top-down control. They reasoned that is has to be top-down because priming is not in the stimulus: “*if we define top-down as guidance of attention by the observer’s knowledge and bottom-up as guidance of attention by the properties of the stimulus, largely independent of that knowledge, then the taxonomy changes. Explicit instruction is clearly top-down, but so is implicit knowledge (whether reliable or not)*” (p. 499). Whether it is implicit or explicit is not immediately relevant for the present discussion. However, what is relevant is that this type of top-down control does not concur with the definitions of what is typically considered to be top-down control. That is, it is neither volitional nor voluntary. It is clear that what has been described by Wolfe et al. (2003) as top-down is a first-class example of the effect of selection history, not malleable by top-down control (see also Failing & Theeuwes, in press for a review).

### 2. Knowing the target guides search in a top-down way

A classic approach to demonstrate top-down guidance of attention is to manipulate whether or not participants know in advance the features of the target they have to search for. A typical method is to have participants search a whole block for one color singleton and another block for a shape singleton. These conditions are then compared to a mixed block in which the target is either (random from trial to trial) a color or a shape singleton creating a condition in which, on each trial, observers do not know the target features they need to search for. With this type of design, Treisman (1988) showed that knowing the dimension of the target (whether it is a color or shape singleton) speeded search by about 100 ms. This was interpreted as evidence for top-down search for feature dimensions. Similar experiments resulting in similar conclusions were conducted, among others, by Found and Muller, (1996), Müller, Heller, and Ziegler (1995) and Wolfe et al. (2003). Even though intuitively this approach seems valid, with these designs it is impossible to determine whether the effects are top-down knowledge-based or merely the result of passive priming (Maljokovic & Nakayama, 1994). Experiments using blocked versus mixed conditions introduce differential intertrial effects and these are considered to be bottom-up in nature (see also Müller et al., 2003 for a similar argument). To make this more explicit: by using a block design in which the target remains the same throughout a whole block of trials, one not only manipulates foreknowledge about the target (which is a top-down set) but one also introduces massive inter-trial priming in which the target of the previous trial primes the target of the current trial. This latter effect is large (e.g., up to 100 ms, see Treisman, 1988) and has nothing to do with top-down processing. All studies that have used a block design (in which the target remains constant throughout a block of trials) to demonstrate how top-down set affects target selection are in this respect invalid (e.g., Müller et al., 1995; Wolfe et al., 2003) as they only have shown that there is intertrial priming. To determine whether there is true top-down volitional guidance, one has to demonstrate its volitional nature implying that one should change the top-down set on a trial-by-trial basis (e.g., one trial search for red, on the next search for green). In other words, one needs to cue on each trial, the feature of the target that participants have to search for. For example, Theeuwes et al. (2006) cued on each trial with 80% validity, the target (search for a shape singleton, or search for color singleton). They showed no cue validity effects under these conditions: the time to find the target did not differ between valid and invalid cue conditions. It was concluded that expectancy-based, top-down knowledge induced by a cue cannot guide the search process. If anything, the cue had only an effect on post-selection processes, i.e., observers were faster to respond after they had selected the target (see Mortier, Theeuwes & Starreveld, 2005).

### 3. Voluntary, bottom-up control

One of the ideas that has been very influential is the notion that bottom-up control is never truly driven by the properties of the environment but always depends on the top-down set of the observer. This notion of voluntary, bottom-up control is known as contingent capture (Folk, Johnston & Remington, 1992). The basic idea is that observers only get captured by a particular stimulus feature (for example a red singleton) when observers are looking for red items. If they are looking for other features (say a singleton presented with abrupt onset) they completely ignore the color singleton. This implies that bottom-up capture is never a “reflex-like” response as originally assumed by Posner (1980) but always dependent on the state of the observer. The original contingent capture finding has been replicated many times, but interestingly enough a crucial aspect of the contingent capture design has always been overlooked. In almost all studies investigating contingent capture, observers are instructed to search for a particular feature singleton (say a color singleton) throughout a whole block of trials. In other words, during the whole block, observers search for color singletons while ignoring abrupt onset singletons. This implies that there will be strong inter-trial effects which in principle have nothing to do with the idea of contingent (top-down) capture (see previous section).

If capture is truly contingent on the top-down set of the observer, one should be able to adjust this top-down set on a trial-by-trial basis in a truly voluntary, volitional way. Remember that this is one of the definitions of top-down control. Belopolsky et al. (2010) did exactly this. They used the same spatial cueing paradigm as Folk et al. (1992), but instead of keeping the target fixed over a whole block of trials (as was originally done with contingent capture experiments), observers needed to adopt their top-down set on the basis of information of a cue presented before the start of each trial. If this capture is truly contingent on top-down set, one expects that only properties that match the cued top-down set would capture attention. Belopolsky et al. showed that even though participants knew what the target would be on the upcoming trial, the irrelevant feature that was not part of the task set also captured attention. There was no evidence for contingent capture: attention was captured in the same amount by relevant and irrelevant singletons. To account for this unexpected result, proponents of contingent capture argued that a top-down set can never be adjusted on a trial-by-trial basis but that one always needs a few trials to “get into the top-down set”. Even though viable, this reasoning basically refutes the idea that true top-down control is even possible and in fact indicates that one needs “selection history” (e.g., “a few trials to get into the top-down set”) to obtain contingent capture like effects.

There has been, however, one study that claimed volitional “contingent capture” control on a trial-by-trial basis. Lien, Ruthruff, and Johnston (2010) used a version of the spatial cueing paradigm of Folk et al. (1992) and cued the color of the upcoming target singleton (i.e., the letter “R” for red and “G” for green) at the center of the display. In one of their experiments, the color to search varied randomly from trial to trial showing a cue validity effect even when the color to look for switched from one trial to the next. This led the authors to conclude that top-down set could be applied in a highly flexible way. Even though on the face of it this seems reasonable, it should be noted that Lien et al. used a spatial cueing paradigm in which selectivity was determined on the basis of cue validity effects. It is likely that the crucial validity effect was primarily driven by the invalid cue conditions resulting from difficulties in disengaging attention from the invalid location since the element at this location had the same color as the target on the previous trial giving rise to inter-trial priming (see also Belopolsky et al., 2010; Theeuwes 2010b for a similar argument). In addition, Lien et al. used only four elements in the display, two of which were color singletons, rendering both singletons non-salient (see also Theeuwes & Van der Burg, 2011).

## What are the differences between top-down and history-based selection?

We only summarize the most important differences between top-down and history-based selection, because there is little dispute over the characteristics of bottom-up selection.

### 1. Slow versus fast

On any given trial, top-down selection is relatively slow, while history-based selection is relatively fast. Faster selection has been shown with studies using reward (Anderson et al., 2011b), statistical learning (Wang & Theeuwes, 2018), priming (Theeuwes & Van der Burg, 2013) and acquired fear (Nissens, Failing & Theeuwes, 2017). Eye movement studies clearly demonstrated the dissociation between fast history-based saccades and relatively slow, top-down saccades (Failing et al., 2015; Nissens et al., 2016; Le Pelley, et al., 2015). Note however, that it should be noted that some studies have reported eye movements that were relatively fast and top-down in origin (Hollingworth, Matsukura & Luck, 2013; Gaspelin, Leonard, & Luck, 2017).

### 2. Automatic versus controlled

Top-down selection is controlling and shifting attention “at will” through space (Posner, 1978, 1980), whereas history-based selection is automatic and basically occurs even when observers try to counteract these effects (Hickey, Chelazzi & Theeuwes, 2010; Theeuwes et al., 2006). Related to this point is that top-down selection is flexible (e.g., can be changed on each trial) while history-based selection is an attentional bias (a bias often unknown to the observer) that cannot be changed in a top-down way. This type of selection has been referred to as “habitual attention” (see Jiang, 2017 for a review).

### 3. Effortless and effortful

Top-down selection requires effort to shift attention. Take for example a Posner cueing task in which a verbal cue points to the likely location of the target; if observers do not actively use and interpret this cue to direct their attention, no top-down cueing effect will be seen. Contrary to this, history-based selection occurs without any effort on part of the observer. In fact, effects of history based selection occur without observers even being aware of stimulus-reward associations (Anderson, 2015; Pearson, Donkin, Tran, Most & Le Pelley, 2015; Le Pelley et al., 2015) stimulus-punishment associations (Hopkins et al., 2016), or statistical regularities in the display (Wang & Theeuwes, 2018; Zhao et al., 2013; Ferrante et al., in press).

## The underlying mechanism of selection history based effects

It is interesting from the analyses above that the properties of history-based selection biases are very similar to bottom-up effects. Just like bottom-up effects, they are fast, automatic, effortless and occur even when observers try to counteract them in a top-down way. For example, a stimulus which acquired value after it was associated with reward continues to capture attention even when observers try to select the target (see Failing & Theeuwes, in press). The continued capture by the stimulus associated with reward implies that top-down set cannot overcome these distraction effects that are very similar to bottom-up saliency capture effects as found with the additional singleton paradigm (Theeuwes, 1991, 1992). However, unlike classic bottom-up effects which are exclusively driven by the salience, selection-history effects occur even when the stimulus associated with reward or punishment is non-salient (Anderson et al., 2011b; Failing & Theeuwes, 2014; Failing et al., 2015; Nissens et al., 2016).

To account for selection history based effects on attention, we assume that the experience with a particular feature (Theeuwes & van der Burg, 2013) or the location of a feature (Wang & Theeuwes, 2018; Ferrante et al., in press) changes its representation within the priority map. In addition to actual physical salience, we adhere to the position that experience with a feature may boost its representation above and beyond its physical salience. For example, intertrial priming may change the salience of a stimulus. Desimone (1996) argued that repeatedly processing a stimulus produces what was called a “sharpening” of its cortical representation, possibly making it more salient within its environment. Bichot and Schall (2002) showed that repeating a stimulus changes responses of neurons in the frontal eye field, a region that has been implicated to be the neural substrate of the salience map (Thompson & Bichot, 2005). Olivers and Hickey (2010) showed that intertrial priming results in latency shifts and amplitude differences in the P1 component of the ERP signal—a signal that is seen 80 to 130 ms following display onset. Clearly an effect so early in time resembles bottom-up like effects. Failing and Theeuwes (2016) showed that reward affects the subjective perception of time. Participants performed temporal oddball task and the results showed that stimuli were perceived to last longer when they signaled a relatively high reward compared to when they signaled no or low reward. Failing and Theeuwes (2016) argued that a stimulus signaling reward is subjectively more salient thereby modulating its attentional deployment and distorting how it is perceived in time. Hickey et al. (2010) concluded that the association with reward changes the representation of a stimulus in early visual areas such that it appears to be more salient than stimuli that have the same physical salience but are not associated with reward. This notion is consistent with the “incentive salience hypothesis” of Berridge and Robinson (1998) which proposes that reward-conditioned stimuli may become salient and attention-drawing because the association with reward changes its perceptual representation (see also Todd & Malanigod, in press) Schneider and Shiffrin.

The similarity between bottom-up control and selection history is also evident in the classic work of (Schneider and Shiffrin 1977; Shiffrin and Schneider, 1977). In their view, an automatic process represents the activation of a sequence of nodes that “*nearly always becomes active in response to a particular input configuration*,” and that “*is activated automatically without the necessity for active control or attention by the subject*” (p. 2). Once the activation is started, the process runs off without the possibility to control it. Shiffrin and Schneider (1977, Experiment 4d) trained observers in a consistently mapped (CM) search task in which observers search for digits among letters. After training, observers had to search for target letters along one diagonal while ignoring items presented along the other diagonal. The results showed that if digits (i.e., former targets) were presented on the-to-be-ignored diagonal they captured attention automatically, even if observers tried to do otherwise. The CM training is a prime example of how selection history biases attention such that, after extensive training, digits presented among letters started to pop out, just like bottom-up capture of a red poppy in a green field. According to Shiffrin and Schneider (1977) controlled (non-automatic) processes are under the control of the person and are established intentionally and volitionally by the person, the same conception regarding top-down control as is adhered to here.

## Conclusion

The model of Awh et al., as displayed in Figure 1, recognizes three selection biases feeding into the integrated priority map. Our message of the current review is that true top-down control of visual selection occurs far less often than what is typically assumed. Most of the time, selection is based on experience and history. This type of selection is fast, automatic and occurs without much effort. Even though this view may appear to be extreme, it fits with the notion that in general, behavior is not controlled in a volitional way, i.e., by actively choosing and controlling actions. Bargh and Chartrand (1999) indicated in their review that our ability to exercise intentional control is in fact quite limited despite the fact that “*much of contemporary psychological research is based on the assumption that people are consciously and systematically processing incoming information in order to construe and interpret their world and to plan and engage in courses of action”* (p. 462).

As stated by William James in his seminal book *The Principles of Psychology* “*each of us literally chooses, by his ways of attending to things, what sort of universe he shall appear himself to inhabit*” James, 1890, p. 424 (James, 1890). It will be clear by now that visual selection as conceived here may not be a deliberate choice as James has put it. Instead “*what sort of universe he shall appear himself to inhabit*” is maybe not a choice but the consequence of our experiences creating a universe that is automatically forced upon us.

## Data Availability

The author has no data accessibility statement to declare.
